# Self-efficacy of medical students in a hybrid curriculum course (traditional and problem-based learning) and associated factors

**DOI:** 10.1186/s12909-023-05016-3

**Published:** 2024-01-03

**Authors:** Marcos Kubrusly, Bianca Oriá Almada de Aquino, Thomás Samuel Simonian, Matheus do Nascimento Oliveira, Hermano Alexandre Lima Rocha

**Affiliations:** 1 Unichristus University Center, R. João Adolfo Gurgel, 133 - Cocó, 60190-180 Fortaleza - CE, Brazil; 2 Community Health Department, Federal University of Ceará, R. Papi Júnior, 1223 - Rodolfo Teófilo, Fortaleza - CE, 60430-235 Fortaleza - CE, Brazil

**Keywords:** Students, Medical, Self efficacy, Problem-based learning, Learning

## Abstract

Self-efficacy consists of the judgment of one’s abilities to perform actions required to achieve a given performance, which has been considered predictive of performance. In academics, it means personal convictions in accomplishing a task to a defined degree of quality. Numerous studies have investigated medical students’ self-efficacy in traditional and PBL curricula. However, few studies have addressed the hybrid PBL scenario (Hpbl) that simultaneously contemplates PBL, traditional teaching, and practical activities. An even smaller number have evaluated the factors associated with this entity. With these considerations, we aimed to investigate the self-efficacy belief in the hPBL curriculum and the factors associated with this entity. This quantitative observational cross-sectional study was held between August 2022 and November 2022 in Fortaleza, a city in Northeast Brazil with almost 3 million inhabitants. The medical course has 12 semesters. The first two semesters use traditional teaching and cover the basic cycle, followed by the third to eighth semesters which correspond to the pre-clinical and clinical cycle. From the third semester onwards, traditional teaching and PBL are used simultaneously, which we call a hybrid model of PBL. The scale “Scale of Self-efficacy in Higher Education” was applied, a questionnaire validated for the Portuguese language consisting of 34 questions, with answers on a Likert-type scale with ten points, divided into five dimensions. To verify the association between sociodemographic factors and self-efficacy, simple and multiple linear models with robust errors were used. In total, 412 students participated in this study, most of them women (70.1%). The average age of students was 22.9 years. All domains had medians greater than 8, which means strong self-efficacy. The following factors were associated with higher self-efficacy scores in general after the multivariate analysis: female gender (8.6 vs. 8.3, *p*-value = 0.014), working (8.8 vs. 8.5, *p*-value = 0.019) and participating in extracurricular activities (8.7 vs. 8.1, *p*-value = 0.019). We conclude that medical students studying in hybrid learning models showed strong levels of self-efficacy. In addition, participating in extracurricular activities was associated with higher self-efficacy scores and males presented lower levels of self-efficacy.

## Introduction

Self-efficacy is a central concept in Bandura’s Social Cognitive Theory because it deals with people’s beliefs about themselves. It belongs to the class of expectations that are linked to the self and represents an important factor in the execution of tasks and in the decisions that subjects will make throughout their lives. It is believed that the greater the perceived self-efficacy, the greater the degree of effort invested and persistence in achieving an established goal. The definition of self-efficacy proposed by Bandura consists of judging one’s abilities to perform actions required to achieve a given performance. That’s because, under this understanding, self-efficacy beliefs have been considered predictive of performance. This association was pointed out by Bandura and confirmed in later studies since it can influence the learning process in the cognitive, motivational, and behavioral aspects and encourage the student to transform psychological skills into school performance skills based on the development of the self-regulation process. In the academic area, the concept does not differ much, as self-efficacy beliefs are personal convictions in accomplishing a task at a defined level of quality [1–4].

It is based on the various studies that converge towards the influence of self-efficacy beliefs on the academic success that the importance of its study in the context of medical education is revealed. University students with strong self-efficacy beliefs can overcome the new behaviors and personal restructuring that this new learning environment requires in a new group with innovative teaching methodologies, which requires a lot from students. This overcoming is partly explained by the fact that students with high self-efficacy are more likely to participate actively, work harder, remain more focused on the problem, and persevere for longer in a situation of coping with a difficult learning task than a student with lower levels of self-efficacy, who are more likely to become frustrated and give up [5–7]. Furthermore, Papinczak et al. (2008) reported a significant association between high self-efficacy and a deeper learning approach. Dias and Azevedo (2001) identified that self-efficacy in higher education is a determinant of academic performance [8, 9].

However, several factors can affect students’ self-efficacy. A previous study in Korea with 244 medical students identified that perfectionism is associated with lower self-efficacy among students [10]. Another study, carried out in Brazil with 147 medical students, found that the teaching methodology is associated with self-efficacy, with greater self-efficacy identified among students exposed to active teaching methodologies [11]. In addition, an Iranian study with 279 students found an association between students’ emotions and self-efficacy, with self-efficacy leading to positive emotions, as proposed by the authors [5].

Numerous studies have investigated medical students’ self-efficacy in traditional and PBL curricula. However, our literature search revealed few studies published in the hybrid PBL scenario (Hpbl), which, at the curricular level, simultaneously contemplates PBL, traditional teaching, and practical activities. In addition, despite the significance of self-efficacy in students’ academic performance, few studies adequately evaluated the construct, and an even smaller number assessed the factors associated with this entity [1]. Therefore, it becomes interesting to assess which factors common to students, such as sociodemographic factors and participation in extracurricular activities, would be implicated in better self-efficacy to develop strategies for better performance of students. With these considerations, we aimed to investigate the self-efficacy belief in the hPBL curriculum and the factors associated with this entity.

## Methods

This is a cross-sectional observational quantitative analytical study.

### Setting and period of study

The study was conducted between August 2022 and November 2022, at Centro Universitário Christus (Unichristus) Campus Parque Ecológico. It is a private Higher Education Institution that offers 20 courses, such as medicine, dentistry, nutrition, psychology, physiotherapy, nursing, etc. The medical course has 12 semesters. The first two semesters use traditional teaching and cover the basic cycle, followed by the third to eighth semesters which correspond to the pre-clinical and clinical cycle. From the third semester onwards, traditional teaching and PBL are used simultaneously, which we call hPBL. The PBL methodology of our course takes place in two four-hour tutorial sessions a week, following the seven steps proposed by Schmidt [12]. Towards the end of the course, in the last four semesters (9 to 12), students do the in-service training cycle (internship) under the supervision of preceptor doctors.

### Population and sampling

The study involved medical students from Unichristus enrolled from the first to the eighth semester.

#### Inclusion criteria

Properly enrolled students who attended at least 75% of classes.

#### Exclusion criteria

Students considered unable to answer the questionnaires due to physical, mental or psychological disability were excluded from the study.

### Data collection

Online questionnaires prepared in Google forms were applied in person. The link to the questionnaire was sent to the students and answered by them digitally on their electronic devices, under the supervision of the researchers.

### Variables

Initially, a sociodemographic and extracurricular activities questionnaire was applied. The variables analyzed were: gender, ethnicity, age, family income, marital status, work, hours of work per week, hours of study per week and participation in extracurricular activities.

After that, the “Self-Efficacy Scale in Higher Education” was applied. This is a questionnaire validated for the Portuguese language, consisting of 34 questions, answered on a Likert-type scale, with ten points, divided into five dimensions. Of the 34 questions that make up the questionnaire, nine assess academic self-efficacy (perceived confidence in the ability to learn, demonstrate and apply knowledge); seven, self-efficacy in training regulation (perceived confidence in the ability to set goals, make choices, plan and self-regulate actions in the process of training and career development); seven, self-efficacy in proactive actions (perceived confidence in the ability to take advantage of training opportunities, update knowledge and promote institutional improvements); seven, self-efficacy in social interaction (perceived confidence in the ability to relate to peers and teachers, for academic and social purposes); and four, self-efficacy in academic management (perceived confidence in the ability to get involved, plan and meet deadlines in relation to academic activities). According to Guerreiro-Casanova and Polydoro (2010), it is possible to categorize self-efficacy into weak (values up to 5.9), moderate (values between 6 and 7.9) and strong (values between 8 and 10). The internal consistency of the scale is 0.94. It varies from 0.80 to 0.81 in the dimensions, and the total explained variance is 56.68, which shows the instrument’s adequacy [13].

### Statistical analysis

The categorical quantitative results were presented in the form of percentages and counts, and the numerical ones in the form of measures of central tendency. Kolmogorov-Smirnov normality tests were performed for numeric variables. To verify the association between sociodemographic factors and self-efficacy, simple and multiple linear models with robust errors were used. In the multiple regressive models, the variables that presented p less than 0.05 in the simple regressive models were included for each of the domains of the self-efficacy scale and for the final result of the scale. The data obtained in the collection were tabulated and analyzed using the IBM SPSS Statistics for Windows software, Version 23.0. Armonk, NY: IBM Corp. IBM Corp. Released 2015.

### Ethical aspects

This project was submitted to the Research Ethics Committee (CEP) of Centro Universitário Christus (Unichristus) under number CAAE 58824522.7.0000.5049. Referencenumber: 5.516.860.

## Results

In total, 412 students participated in this study, most of which were women (70.1%). The average age of students was 22.9 years old, with a standard deviation of 5.8, and most were white (71.4%). More than 80% of students reported family income higher than 5 Brazilian minimum wages (one salary represented US$ 231.96 at the time of data collection in 2022), but 8.4% received up to 3 minimum wages of family income or less than 10 dollars per capita per day in a family of 3 people. More than 90% of students only study, and those who work are reported to work an average of 1.6 h a week. The average weekly time dedicated to studies by students was 30.2 h, and 11.7% of students were married. Most respondents were in the first semester (27.1%), followed by the second semester (19. 6%) and the fourth (22%). Finally, 70.5% of the students carried out extracurricular activities. The descriptive data of the evaluated sample can be seen in Table 1 (Table 1).

**Table 1:** Description of study participants. (N = 412)

		N (%) or average (SD)
What’s your gender?		
	Feminine	289 (70.1)
	Masculine	123 (29.9)
How old are you? (in years, mean and SD)		22.9 (5.8)
What is your ethnicity?		
	White	295 (71.4)
	Black	5 (1.2)
	Brown	113 (27.4)
What is your family income?		
	Up to 1 salary	8 (1.9)
	From 1 to 3 salaries	27 (6.5)
	From 4 to 5 salaries	36 (8.7)
	More than 5 salaries	343 (82.9)
What is your occupation?		
	I just study	377 (91.1)
	Work and study	37 (8.9)
If so, how many hours do you work per week?		1.6 (6.3)
How many hours do you study per week?		30.2 (18.3)
What is your marital status?		
	Married	47 (11.7)
	Single	354 (88.3)
What is your course?		
	Medicine	414 (100)
What’s your semester?		
	s1	112 (27.1)
	s2	81 (19.6)
	s3	41 (9.9)
	s4	91 (22.0)
	s5	1 (0.2)
	s6	20 (4.8)
	s7	13 (3.1)
	s8	55 (13.3)
Do you participate in extracurricular activities?		
	No	122 (29.5)
	Yes	292 (70.5)

The average results in each self-efficacy domain can be seen in Table 2. All domains had medians greater than 8, which means strong self-efficacy. The 25th percentile of all domains was also greater than 7.5 (less for the domain of proactive action). The proactive actions domain also had the lowest minimum value among students (Table 2). By descriptively analyzing the results of the individual items that make up each of the self-efficacy domains, we see that all items in all domains had medians from 8 onwards, indicating strong self-efficacy. However, some domains showed results consistently close to 8 and no 10, such as academic self-efficacy and self-efficacy in training regulation (the latter did not show any item even with 9), while the other 3 had higher medians for their individual items.

**Table 2:** Results of measures of central tendency and dispersion of domains and overall self-efficacy result

	median	25th percentile	75th percentile	Minimum	Maximum
Academic effectiveness	8.44	7.89	9.11	3.67	10.00
Training regulation	8.57	7.71	9.14	3.29	10.00
Proactive actions	8.07	7.29	8.86	3.14	10.00
Social interaction	8.86	8.00	9.43	4.71	10.00
Academic management	9.00	8.50	9.75	5.75	10.00
General self-efficacy	8.56	7.82	9.06	4.76	10.00

In Table 3, we see the factors that were associated with the domains of academic self-efficacy, self-efficacy in regulating education and self-efficacy in proactive actions. After multivariate analysis, participation in extracurricular activities was statistically associated with greater academic self-efficacy (8.6 vs. 8.1, *p* < 0.001); female gender and participation in extracurricular activities were associated with higher scores on self-efficacy in regulation (8.6 vs. 8.3, *p*-value = 0.015 and 8.6 vs. 8.3, *p*-value < 0.001, respectively) ; and, finally, female gender and participation in extracurricular activities were also associated with higher scores in self-efficacy in proactive actions, with men and those who did not participate presenting scores one whole degree lower than women in this domain (8.1 vs. 7, 7, *p* value = 0.002 and 8.1 vs. 7.4, *p* value < 0.001, respectively) (Table 3).

**Table 3:** Factors associated with the domains academic effectiveness, training regulation and proactive actions

		Academic effectiveness			Training regulation			Proactive actions		
		Median (CI 95)	*p*-value	p* value	Median (CI 95)	*p*-value	p* value	Median (CI 95)	*p*-value	p* value
What’s your gender?			0.243			**0.012**	**0.015**		**0.002**	**0.005**
	Feminine	8.6 (8.6–8.8)			8.6 (8.4–8.9)			8.1 (8.1–8.3)		
	Masculine	8.3 (8.1–8.6)			8.3 (8-8.7)			7.7 (7.4-8)		
	Total	8.4 (8.4–8.6)			8.6 (8.4–8.7)			8.1 (8.1–8.3)		
How old are you? (Categorized)			0.158			**0.034**	0.187		**0.003**	0.14
	<= 19	8.6 (8.4–8.8)			8.6 (8.4–8.7)			8.1 (8-8.4)		
	20–22	8.3 (8.1–8.6)			8.3 (8-8.7)			7.9 (7.6–8.1)		
	23+	8.4 (8.3–8.7)			8.6 (8.4–8.9)			8.2 (8.1–8.4)		
	Total	8.4 (8.4–8.6)			8.6 (8.4–8.7)			8.1 (8-8.1)		
What is your ethnicity?			0.198			0.088			0.199	
	White	8.4 (8.3–8.6)			8.6 (8.4–8.7)			8.1 (8-8.3)		
	black	9 (8.8–9.8)			9.1 (8.6–10)			8.3 (7.6–9.3)		
	Brown	8.4 (8.3–8.7)			8.3 (8.1–8.7)			7.9 (7.6–8.1)		
	Total	8.4 (8.4–8.6)			8.6 (8.4–8.7)			8 (8-8.1)		
What is your family income?			**0.032**	0.113		0.18			0.918	
	Up to 1 salary	8.4 (7.3–9.2)			8 (7.4–8.6)			7.8 (7.3-9)		
	From 1 to 3 salaries	8.4 (8.2–8.9)			8.4 (8.1–9.1)			8.3 (7.9–8.6)		
	From 4 to 5 salaries	7.9 (7.7–8.6)			8.2 (7.9–8.6)			8 (7.7–8.4)		
	More than 5 salaries	8.4 (8.4–8.6)			8.6 (8.4–8.7)			8.1 (8.1–8.3)		
	Total	8.4 (8.4–8.6)			8.6 (8.4–8.7)			8.1 (8-8.1)		
What is your occupation?			0.052			**0.06**			**0.04**	0.086
	I just study	8.4 (8.4–8.6)			8.6 (8.4–8.7)			8 (8-8.1)		
	Work and study	8.9 (8.4–9.3)			8.7 (8.4–9.3)			8.4 (8-9.1)		
	Total	8.4 (8.4–8.6)			8.6 (8.4–8.7)			8.1 (8-8.1)		
What is your marital status?			0.889			0.535			0.426	
	Married	8.4 (8.2–8.7)			8.7 (8.4–9.3)			8.1 (8-8.7)		
	Single	8.4 (8.4–8.6)			8.6 (8.4–8.7)			8 (8-8.3)		
	Total	8.4 (8.4–8.6)			8.6 (8.4–8.7)			8 (8-8.1)		
What’s your semester?			0.058			0.23			0.22	
	s1	8.4 (8.2–8.6)			8.3 (8.1–8.7)			8 (7.7–8.4)		
	s2	8.7 (8.4–8.9)			8.7 (8.4-9)			8.1 (8-8.6)		
	s3	8.3 (8.1–8.9)			8.4 (7.9–8.9)			8 (7.6–8.3)		
	s4	8.6 (8.6–8.9)			8.6 (8.4-9)			8.1 (8-8.4)		
	s5	6.8 ((.)-(.))			8.9 ((.)-(.))			6.6 ((.)-(.))		
	s6	8.7 (8.1–9.3)			8.5 (7.4-9)			7.4 (6.3–8.4)		
	s7	8.3 (7.9–8.8)			8.1 (7.9–8.9)			7.6 (7.3–8.3)		
	s8	8.6 (8.3-9)			8.6 (8.4–9.1)			8.3 (7.9–8.7)		
	Total	8.4 (8.4–8.6)			8.6 (8.4–8.7)			8.1 (8-8.1)		
Do you participate in extracurricular activities?			**< 0.001**	**< 0.001**		**0.001**	**< 0.001**		**< 0.001**	**< 0.001**
	No	8.1 (8-8.4)			8.3 (8-8.7)			7.4 (7.3-8)		
	Yes	8.6 (8.4–8.8)			8.6 (8.4–8.9)			8.1 (8.1–8.3)		
	Total	8.4 (8.4–8.6)			8.6 (8.4–8.7)			8.1 (8-8.1)		

* After multivariate adjustment

Factors associated with the remaining two domains, self-efficacy in social interaction and self-efficacy in academic management and general self-efficacy, can be seen in Table 4. After multivariate analysis, it was found that working is associated with greater self-efficacy in social interaction, as well as participating in extracurricular activities (9.3 vs. 8.7, *p*-value = 0.003 and 9.0 vs. 8.4, *p*-value < 0.001, respectively); that again the female sex and participating in extracurricular activities are positive, this time in the domain of self-efficacy in academic management (9.3vs 9.0, *p* value = 0.001 and 9.3 vs. 8.8, *p* value < 0.001, respectively) (Table 4).

The main result of the study can also be seen in Table 3. Female gender (8.6 vs. 8.3, *p*-value = 0.014), working (8.8 vs. 8.5, *p*-value = 0.019) and participating in extracurricular activities (8.7 vs. 8.1, *p* value = 0.019) were associated with higher self-efficacy scores overall after multivariate analysis (Table 4).

**Table 4:** Factors associated with the domains self-efficacy in social interaction, self-efficacy in academic management and general self-efficacy

		Social interaction			academic management			general self-efficacy		
		Median (CI 95)	*p*-value	p* value	Median (CI 95)	*p*-value	p* value	Median (CI 95)	*p*-value	p* value
What’s your gender?			0.143			**0.002**	**0.001**		**0.011**	**0.014**
	Feminine	8.9 (8.7-9)			9.3 (9.3–9.5)			8.6 (8.6–8.8)		
	Masculine	8.7 (8.6-9)			9 (9-9.5)			8.3 (8.1–8.6)		
	Total	8.9 (8.7-9)			9 (9-9.3)			8.6 (8.4–8.6)		
How old are you? (Categorized)			< 0.001			**0.028**	0.888		**0.005**	0.545
	<= 19	8.9 (8.7-9)			9.3 (9.3–9.5)			8.6 (8.4–8.8)		
	20–22	8.4 (8.3–8.7)			9 (9-9.3)			8.3 (8.1–8.6)		
	23+	9 (8.9–9.3)			9.3 (9.3–9.5)			8.6 (8.4–8.8)		
	Total	8.9 (8.7-9)			9 (9-9.3)			8.6 (8.4–8.6)		
What is your ethnicity?			0.387			0.092			0.079	
	White	8.9 (8.7-9)			9.3 (9.3–9.5)			8.6 (8.6–8.8)		
	Black	8.9 (8.9–10)			9.5 (9.5–10)			8.9 (8.4–9.8)		
	Brown	8.7 (8.6-9)			9 (9-9.3)			8.4 (8.2–8.6)		
	Total	8.9 (8.7-9)			9 (9-9.3)			8.6 (8.4–8.6)		
What is your family income?			0.112			0.418			0.126	
	Up to 1 salary	8 (7.6–9.7)			9 (7.8–10)			8.3 (7.4-9)		
	From 1 to 3 salaries	8.7 (8.4–9.3)			9 (9-9.3)			8.6 (8.1–8.9)		
	From 4 to 5 salaries	8.4 (8.1-9)			9 (9-9.5)			8.2 (7.9–8.6)		
	More than 5 salaries	8.9 (8.7-9)			9.3 (9.3–9.5)			8.6 (8.4–8.7)		
	Total	8.9 (8.7-9)			9 (9-9.3)			8.6 (8.4–8.6)		
What is your occupation?			**0.003**	**0.003**		0.098			**0.015**	**0.019**
	I just study	8.7 (8.7-9)			9 (9-9.3)			8.5 (8.4–8.6)		
	Work and study	9.3 (9-9.7)			9.5 (9-9.8)			8.8 (8.6–9.4)		
	Total	8.9 (8.7-9)			9 (9-9.3)			8.6 (8.4–8.6)		
What is your marital status?			0.264			0.705			0.244	
	Married	9 (8.7–9.4)			9 (9-9.5)			8.6 (8.3–8.9)		
	Single	8.9 (8.7-9)			9.3 (9.3–9.5)			8.6 (8.4–8.7)		
	Total	8.9 (8.7-9)			9 (9-9.3)			8.6 (8.5–8.7)		
What’s your semester?			0.33			0.639			0.243	
	s1	9 (9-9.3)			9.1 (9-9.3)			8.4 (8.2–8.8)		
	s2	8.7 (8.4–9.1)			9.3 (9.3–9.8)			8.6 (8.3–8.8)		
	s3	8.7 (8.3-9)			9 (8.8–9.5)			8.4 (8.1–8.8)		
	s4	8.7 (8.6–9.1)			9 (9-9.5)			8.6 (8.5–8.8)		
	s5	6.6 ((.)-(.))			9.5 ((.)-(.))			7.4 ((.)-(.))		
	s6	8.8 (7.9–9.3)			8.8 (8.5–9.8)			8.5 (7.6–9.1)		
	s7	8.3 (8.1–9.3)			8.5 (8-9.8)			8.1 (7.7–8.9)		
	s8	9 (8.7–9.3)			9 (8.8–9.8)			8.7 (8.4-9)		
	Total	8.9 (8.7-9)			9 (9-9.3)			8.6 (8.4–8.6)		
Do you participate in extracurricular activities?			**< 0.001**	**< 0.001**		**< 0.001**	**< 0.001**		**< 0.001**	**< 0.001**
	No	8.4 (8.3–8.9)			8.8 (8.5-9)			8.1 (7.8–8.4)		
	Yes	9 (9-9.3)			9.3 (9.3–9.5)			8.7 (8.6–8.8)		
	Total	8.9 (8.7-9)			9 (9-9.3)			8.6 (8.4–8.6)		

* After multivariate adjustment

## Discussion

In this study, we identified that medical students in hybrid curricula had a strong level of self-efficacy. Being female, participating in extracurricular activities, and working were associated with higher self-efficacy scores.

PBL was first introduced at McMaster University in the late 1960s and was later widely accepted by medical schools worldwide [14]. Simultaneously, several schools suggested modifications to the original PBL format and advocated for alternative approaches that led to the birth of “hybrid” PBL (hPBL). [15]. Havard’s New Pathway curriculum has altered the scope, frequency, and format of its dialogue lectures and hands-on lab classes and hybridized them with problem-based active discussion (PBL). [16]. This is our institution’s model since, like Malik, [15] we believe that this model has the advantages of reducing knowledge gaps, establishing a solid foundation for education in fundamental disciplines, and covering different learning styles, among others. However, as students in this model have the obligation, in addition to the PBL activities, to simultaneously participate in dialogued expositions and practical exercises for about 10 h a week, a higher cognitive overload is observed in relation to the “pure” PBL model. a fact that leads these students to an important degree of resilience when compared to other teaching models [15, 17].

This burden was somewhat confirmed during the pandemic, where we found that the prevalence of zoom fatigue reached 56% in students using the hybrid model, against 41% using the PBL methodology, a statistically significant difference (*p* value = 0.027) [18]. Faced with the hPBL, we question whether this curricular model would be able to influence the development of self-efficacy, that is, the students’ conviction (self-efficacy) in meeting the required academic demands. The present study showed that all items in all domains had medians of eight or more, indicating strong self-efficacy. These results are comparable with the results of self-efficacy found for the PBL methodology (mean of 8.1 ) and are higher than the results found in traditional methodology (mean 7.5) in the study carried out by Lopes et al. in two medical schools [19]. This result can partly be explained by the fact that problem-based learning belongs to the hPBL curriculum since active teaching methodologies affect self-efficacy factors: personal experiences, vicarious experiences, social persuasion, and physiological indicators. Activities based on problem-solving, simulations, and project development can lead to positive personal experiences and increased personal belief in the ability to perform a given task [20, 21]. In addition, hPBL curriculum students have a greater academic demand than other curricula because they have to simultaneously acquire knowledge from traditional classes and practical activities and actively seek knowledge when using problem-based learning methodology. These demands, as we have already demonstrated, lead to an important degree of resilience in this curriculum model which, in turn, presents, according to the literature, a positive effect on students’ self-efficacy and, consequently, demands greater effort by students and also leads to the development of learning self-regulation [17, 22, 23].

It is noteworthy that in addition to the active methodology and resilience, the traditional teaching model itself can equally contribute to the development of self-efficacy in the hPBL curriculum. Corroborating this assumption, a longitudinal design study conducted by Schauber et al. [21] evaluated the self-efficacy of 1,646 students, from the 6th to the 10th semester, in a traditional curriculum or one centered on problem-based learning at the University of Medicine Charité in Berlin, Germany. After analyzing the data, Schauber et al. verified the inexistence of substantial differences between the traditional curriculum and the APB in relation to psychosocial variables and performance. They further reported that, in both contexts, gains in performance were related to self-reported study efforts. In conclusion, curriculum reforms do not seem to necessarily deliver the intended benefits compared to more traditional learning environments, as students develop self-efficacy and make substantial efforts to achieve their goals and succeed in their studies anyway, depending on the study context carried out. They emphasized, however, that such an inference does not mean that any change or curriculum reform is inevitably unnecessary, indicating to focus on the following questions: “how”, “why”, “which” and “when” a specific content needs to be acquired by students to choose and allocate resources that facilitate the acquisition of specific knowledge, skills, and competencies adequately and efficiently [21]. It’s important to note that the findings of this study are comparable to our results for students in the 3rd to 8th semesters, who are in APB.

The results of the present study revealed that female gender, extracurricular activities, and working, that is, having a paid job, were the main factors related to higher self-efficacy scores in general after the multivariate analysis. Several studies in the literature show that women are more proactive and capable of planning and executing academic activities; others show predominance in males. This divergence is possibly because there are differences in the population sample of the studies, such as the number of participants and other socio-cultural factors [24, 25]. Studies have reported that women are likelier to use specific learning strategies with appropriate goal structuring [26, 27]. Pajares (2002) reported that women showed more goal-setting and planning strategies, kept records, and often structured their environment for optimal learning [28]. Corroborating with the reports above, Ommering et al. showed that college women were significantly more confident in taking additional notes, planning time for exams, asking friends for help, participating in academic discussions with friends, and understanding feedback on assignments, and paying attention during lectures [29].

Concerning the positive relationship between extracurricular activities (EA) and observed self-efficacy, it can be explained by the fact that these activities allow students to expand their social networks, express and explore their identity, learn new skills, and develop personal qualities, such as soft skills and leadership skills, that have positive effects on academic self-efficacy performance [23, 30, 31]. Corroborating the above report, Santos and Fior and Mercuri, describe that extracurricular activities can stimulate the development of university student characteristics in five main domains: (1) academic knowledge and skills, (2) cognitive complexity, (3) practical competence, (4) Interpersonal competence, and (5) Humanitarianism, which can explain its relationship with the domain of self-efficacy in training regulation, proactive actions, social interaction as well as academic management [32]. In summary, the benefits of non-compulsory experiences are reflected in several aspects, which can help the student develop in these various areas and lead to a better use of the course. In the hPBL curriculum, we consider self-efficacy (self-regulation, academic management, proactive actions) necessary to extracurricular activities at our institution since students have to organize their time to carry them out due to the great academic demand. They also feel obliged to do so, as they are important in the analysis of medical residency exam in Brazil [33].

As reported, another factor related to higher overall self-efficacy scores after multivariate analysis in our study was working off-campus during medical school. The ability to balance work, full-time study, and social life have become a pervasive trait for many college students, many of whom have to work out of financial necessity [34]. Although not recorded in the present study, this is also the motivation for our students’ work. In Brazil, the annual average amount to be paid in a medical course is 28 thousand dollars, which corresponds to 154 minimum wages in the country. Therefore, among other things, they must be resilient to reconcile work with full-time study and consequently develop self-efficacy. In this context, Gbadamosi et al. conclude in their research that working significantly predicts self-efficacy in the sample of students analyzed. This strong connection demonstrates that working students also have greater self-esteem and self-confidence [35]. In addition, working reduces financial stress that positively impacts student’s well-being, which implies self-acceptance, positive relationships with others, a sense of autonomy, competence, goal setting, and a focus on personal growth that, consequently, leads to the development of self-efficacy [36–38]. These factors explain the positive relationship between working students and self-efficacy. With regard to the specific relationship of the student who works with the domain of social interaction, it can be explained by the fact that work is an important component of life, not only in the material sense (salary, benefits) but also concerning the performance of activities and for social interaction [38]. Therefore, we believe that the work experience and the feeling of well-being that comes from it leads the student to perceive and acquire confidence in his ability to relate to others, contributing to academic adequacy and success. According to Basso, Graf, Lima, Schmidt and Bardagi (2013), the university’s adaptation and success are related to curricular aspects and how the student develops and gives meaning to the interpersonal relationships built in the academic context [39].

This study had some limitations. First, as this is a cross-sectional study, the associated factors we found cannot be defined as causal. Furthermore, although we used a validated self-efficacy scale, it is not exhaustive of the occurrence of self-efficacy in each individual, even though it has shown good accuracy in the studies that tested it. In addition, we didn’t include students from the last four semesters who were doing their internship, and the fifth semester of the course was underrepresented in the sample. Moreover, as the study was carried out in a single center, the results found here may not be generalizable to all medical schools. Finally, adherence sampling may have introduced bias in the selection of students.

## Conclusions

We conclude that hybrid learning models, using traditional and active methodologies, lead to strong levels of self-efficacy in medical students, higher than that of studies with students in the exclusively traditional model. In addition, extracurricular activities were associated with a higher self-efficacy score, which suggests that actions to encourage student participation in EA can be beneficial. Also, as males had lower levels of self-efficacy, they may benefit more from efforts in this regard.

## Data Availability

The datasets used and/or analyzed during the current study are available from the corresponding author upon reasonable request.

## Abbreviations

PBL: Problem-Based Learning
EA: Extracurricular activities
hPBL: Hybrid Problem-Based Learning

## References

[1] Klassen RM, Klassen JRL (2018). Self-efficacy beliefs of medical students: a critical review. Perspect Med Educ.

[2] Bandura A. Social foundations of thought and action: A social cognitive theory. In: Social foundations of thought and action: A social cognitive theory. Prentice-Hall, Inc; 1986: xiii, 617-xiii.

[3] Bandura A (2001). Social cognitive theory: an agentic perspective. Annu Rev Psychol.

[4] Bandura A (1977). Self-efficacy: toward a unifying theory of behavioral change. Psychol Rev.

[5] Hayat AA, Shateri K, Amini M, Shokrpour N (2020). Relationships between academic self-efficacy, learning-related emotions, and metacognitive learning strategies with academic performance in medical students: a structural equation model. BMC Med Educ.

[6] Costa Filho JO, Murgo CS, Franco AF. SELF-EFFICACY IN MEDICAL EDUCATION: A SYSTEMATIC REVIEW OF LITERATURE. SciELO Preprints. 2021.

[7] Bhati K, Baral R, Meher V (2022). Academic self-efficacy and academic performance among undergraduate students in relation to gender and streams of Education. Indonesian J Contemp Educ.

[8] Papinczak T, Young L, Groves M, Haynes M (2008). Effects of a metacognitive intervention on students’ approaches to learning and self-efficacy in a first year medical course. Adv Health Sci Educ Theory Pract.

[9] Dias GF, Azevedo M. Desenvolvimento psicológico, atitudes em relação ao estudo e sucesso acadêmico. Fases on-line: factores de sucesso/insucesso do ensino superior. 2001;1.

[10] Yu JH, Chae SJ, Chang KH (2016). The relationship among self-efficacy, perfectionism and academic burnout in medical school students. Korean J Med Educ.

[11] Lopes JM, Castro JGF, Peixoto JM, Moura EP. Self-efficacy of medical students in two schools with different education methodologies (problem-Basead learning versus traditional). Revista Brasileira De Educação Médica. 2020;44(2).

[12] Schmidt HG (1983). Problem-based learning: rationale and description. Med Educ.

[13] Polydoro SAJ, Guerreiro-Casanova DC (2010). Escala De auto-eficácia na formação superior: construção e estudo de validação. Avaliação Psicológica.

[14] Neville AJ, Norman GR (2007). PBL in the undergraduate MD program at McMaster University: three iterations in three decades. Acad Med.

[15] Malik AS, Malik RH (2018). What really is Hybrid Problem-based learning curriculum? A review. Quest Int J Med Health Sci.

[16] Armstrong EG. A hybrid model of problem-based learning. The challenge of problem-based learning. Routledge; 2013. pp. 145–58.

[17] Kubrusly M, Rocha HAL, Maia ACC, Sá AKDM, Sales MM, Mazza SR (2019). Resilience in the training of medical students in a University with a hybrid teaching-learning system. Revista Brasileira De Educação Médica.

[18] de Sobral OK, Lima JB, Lima Rocha DLF, de Brito HA, Duarte ES, Bento LHG (2022). Active methodologies association with online learning fatigue among medical students. BMC Med Educ.

[19] Melo Prado H, Hannois Falbo G, Rodrigues Falbo A, Natal Figueirôa J (2011). Active learning on the ward: outcomes from a comparative trial with traditional methods. Med Educ.

[20] Bandura A (1997). Self-efficacy: the exercise of control.

[21] Schauber SK, Hecht M, Nouns ZM, Kuhlmey A, Dettmer S (2015). The role of environmental and individual characteristics in the development of student achievement: a comparison between a traditional and a problem-based-learning curriculum. Adv Health Sci Educ.

[22] Siddique M, Ali M, Nasir N, Awan TH, Siddique A (2021). Resilience and Self-Efficacy: a correlational study of 10th Grade Chemistry students in Pakistan. Multicultural Educ.

[23] Hayat AA, Choupani H, Dehsorkhi HF (2021). The mediating role of students’ academic resilience in the relationship between self-efficacy and test anxiety. J Educ Health Promot.

[24] Ranasinghe P, Wathurapatha WS, Mathangasinghe Y, Ponnamperuma G (2017). Emotional intelligence, perceived stress and academic performance of Sri Lankan medical undergraduates. BMC Med Educ.

[25] Partido BB, Stafford R (2018). Association between Emotional Intelligence and Academic Performance among Dental Hygiene Students. J Dent Educ.

[26] Zimmerman BJ, Martinez-Pons M (1990). Student differences in self-regulated learning: relating grade, sex, and giftedness to self-efficacy and strategy use. J Educ Psychol.

[27] Ray MW, Garavalia LS, Gredler ME. Gender Differences in Self-Regulated Learning, Task Value, and Achievement in Developmental College Students. 2003.

[28] Pajares F. Overview of Social Cognitive Theory and Self-efficacy,[Online] Available: http://www.emory.edu.EDUCATION/mfp/eff.html. 2002.

[29] Ommering BWC, van Blankenstein FM, van Diepen M, Dekker FW (2021). Academic success experiences: promoting Research Motivation andself-efficacy beliefs among medical students. Teach Learn Med.

[30] Alhadabi A, Karpinski AC (2020). Grit, self-efficacy, achievement orientation goals, and academic performance in University students. Int J Adolescence Youth.

[31] Feraco T, Resnati D, Fregonese D, Spoto A, Meneghetti C (2022). Soft skills and extracurricular activities sustain motivation and self-regulated learning at school. J Experimental Educ.

[32] Fior CA, Mercuri E. Formação universitária e flexibilidade curricular: importância das atividades obrigatórias e não obrigatórias. Psicologia da Educação. 2009:191–215.

[33] Santos L, Almeida LS (2000). Vivências académicas e rendimento escolar: estudo com alunos universitários do 1º ano. Instituto De Educação E Psicologia.

[34] Pregoner JD, Accion N, Buraquit D, Amoguis A. The Experiences of working while studying: A phenomenological study of senior high school students. 2020.

[35] Gbadamosi G, Evans C, Obalola MA (2016). Multitasking, but for what benefit? The dilemma facing Nigerian university students regarding part-time working. J Educ Work.

[36] Dyrbye LN, Power DV, Massie FS, Eacker A, Harper W, Thomas MR (2010). Factors associated with resilience to and recovery from burnout: a prospective, multi-institutional study of US medical students. Med Educ.

[37] Costa Filho JO, Murgo CS, Franco AF, INFLUENCE OF SELF-EFFICACY AND, SUBJECTIVE, WELL-BEING IN. THE LEARNING OF MEDICINE STUDENTS. SciELO Preprints. 2022.

[38] Creed PA, French J, Hood M (2015). Working while studying at university: the relationship between work benefits and demands and engagement and well-being. J Vocat Behav.

[39] Basso C, Graf LP, Lima FC, Schmidt B, Bardag MP (2013). Organização De tempo e métodos de Estudo: Oficinas com estudantes universitários. Revista Brasileira De Orientação Profissional.

